# Second language learners and the variable speech signal

**DOI:** 10.3389/fpsyg.2014.01338

**Published:** 2014-11-20

**Authors:** Christine E. Shea

**Affiliations:** Department of Spanish and Portuguese, University of IowaIowa City, IA, USA

**Keywords:** allophones, second language acquisition, phonetic variability, bilingualism, L2 phonology

In the article “Age of acquisition and allophony in Spanish-English bilinguals” Barlow (2014) presents production data of /l/ from two groups of Spanish-English bilinguals, who differ on age of acquisition of English (before 5 years or after 6 years of age). Barlow's contribution is a welcome addition to the relatively understudied field of allophone acquisition by second language learners. In what follows I expand upon issues touched upon by Barlow in her article and comment more generally on why the issue of variability in the speech stream (of which allophones in complementary distribution is but one type) must be addressed differently for L2 learners than for infants acquiring a first language. I restrict the discussion to perception and will primarily address issues key to adult (i.e., individuals who began to acquire their second language after the sound system of their first language is in place) second language acquisition.

Research on how adults perceive non-native sounds has received considerable attention over the past thirty years (see work by Flege and Best for the most influential models of L2 perception) and the vast majority of this work has looked at the way in which non-native sounds assimilate into native-language sound categories, independent of the context in which they occur (for an exception, see Levy and Strange, 2008). In a certain sense, it can be said that much of this research abstracts away from speech perception as it unfolds in real time (McMurray and Jongman, 2011). Part of the challenge real-time speech perception represents for L2 learners involves dealing with the way co-occurring sounds (or abstract contexts such as stress, see Shea and Curtin, 2011) affect each other or lead to variability, whether predictable or indexical in nature.

The study of allophone acquisition represents an effort to break away from this tradition and can be included in the broader research program that examines how learners deal with the variability found in the input. Indeed, variability itself is “highly variable” and can be due to individual speaker differences, dialect differences, speech rate, and formality. These kinds of variability are often distinguished from allophonic variability that is the result of phonetic or phonological factors and tend to occur in a more across-the-board fashion in speech.

In terms of L1 acquisition, part of learning a language's sound system necessarily involves learning which sounds contrast and which do not. Research suggests that distributional knowledge and phonetic similarity play a key role in guiding infants toward identifying non-phonemic sounds in their language (see Seidl and Cristia, 2012 for an excellent overview; see Yeung and Werker, 2009, for work showing that a lack of lexical contrast can be used by infants to acquire allophones in non-contrastive distributions as well). For example, in a recent study, Seidl et al. (2009) examined the role of phonemic vs. allophonic contrasts in infant speech perception. They familiarized French-learning 11-month-old and English-learning 11- and 4-month-old infants to syllables in which the final consonants conditioned the nasality of the previous vowel. In French, nasality is phonemic while in English it is allophonic. The results showed that French-learning 11-month-olds and English-learning 4-month- olds had a reliable pattern of preference while English 11-month-olds were insensitive to the patterning, orienting equally to syllables following and violating the familiarized patterns. The authors conclude that language-specific sensitivity to context-driven allophonic contrasts emerges as early as 11 months of age.

In contrast, adult native listeners distinguish allophonic contrasts at a phonetic level less accurately than phonemic contrasts. For example, Pegg and Werker (1997), using an AX discrimination task, showed that native English-speaker adults' performance on the allophonic contrast between voiced [d] and the voiceless unaspirated [t] was better than chance, but nonetheless worse than that on a phonemic contrast (for similar results see Whalen et al., 1997).

In addition to perceiving the difference between two different phones, there is another important component to allophonic acquisition: its context-driven nature. Specifically, allophonic perception cannot be truly categorized as such unless the sounds occur in the context in which they are expected (or not, see Shea and Curtin, 2011 for details; Key, 2014). For example, Peperkamp et al. (2001), using the French [χ] - [ʁ] alternations showed that French listeners could discriminate between allophonic segments in CV syllables but as soon as the CV syllables were put into their allophonic contexts, such discrimination disappeared. Thus, to truly speak of “allophone perception” listeners must be aware of the contrast but also the context in which it occurs.

The mechanism by which infants build their sound categories is based upon tracking distributional frequencies across the speech stream (Maye et al., 2002). A number of laboratory studies reveal that such learning is possible in both infants and adults (Maye and Gerken, 2001; Hayes-Harb, 2007). Nonetheless, a recent study by Wanrooij et al. (2014) suggests that there may be differences between infants and adults in terms of the capacity each group has for making use of distribution-based learning. Wanrooij et al. use MMN imaging and the odd-ball paradigm to show that Dutch infants can be trained on a bimodal distribution to distinguish non-Dutch vowels whereas adult learners do not show such sensitivity. This suggests that a distribution-based learning mechanism is indeed weaker in adults than in infants.

For adult second language learners, neither phonetic similarity nor distributional knowledge is necessarily available for allophone acquisition. Distributions may be objectively present in the speech stream but adult L2 listeners will not necessarily perceive them in a faithful fashion (see extensive work by Flege and colleagues on how L2 speech categorization may be impeded, depending upon the phonetic proximity of the target sound to native sound categories). Thus, the raw input that infants use to create their phonetic categories does not get processed in the same way by adult L2 learners and as a consequence, phonetic similarity is also judged differently: two sounds that are similar to native ears may not be at all similar to L2 ears. This raises questions regarding how the input is processed and stored by adult second language learners in the creation of these new categories.

All is not lost for adults, however. While distribution-based learning relies upon implicit learning mechanisms, adult learners (as compared to infant and child learners) can use explicit learning mechanisms to at least become aware of allophonic alternations. Whether in the second language classroom or in naturalistic learning contexts, adults can be taught where to expect variability in their target language or they can express an explicit awareness when exposed to it. This does not mean that production/perception will necessarily follow, but it does mean that the adult learner can be explicitly aware of an alternation that infants must acquire implicitly and this explicit awareness may serve to initiate perceptual tuning to the L2 input.

For literate learners, spelling is another factor that may influence how variability is processed. Many L2 learners acquire the target language in classroom contexts where, from the first day of class, they are encouraged to read and write in their second language. Thus, target language literacy begins prior to the establishment of phonological and phonetic categories and may result in an overreliance on L1 sound-spelling correspondences, particularly at the earliest stages of L2 acquisition. In the case of allophones, this may be especially problematic. Allophones that belong to the same category often share an orthographic symbol that corresponds to the phonemic category. The shared orthographic symbol encourages the learner to ignore the phonetic variants in the input and build one category for both allophones. Orthography may also hinder the development of L2 allophonic categories when native language allophones correspond to different orthographic symbols in the target language, inadvertently encouraging the learner to think they need to create a new category all together. An example of this latter situation occurs with the flap in English (as in “water”) and the tap in Spanish (as in “pero”). These two sounds are acoustically and articulatorily very similar but in English the flap is an allophone while in Spanish, the tap is a phoneme. In spite of their similarity along acoustic and articulatory dimensions, the sounds are represented by different orthographic symbols in each language, hindering recognition and encouraging the creation of a totally new category. In sum, orthography can help or hinder the acquisition of allophones in a second language, depending upon the L1-L2 categories involved.

Another issue related to input is whether the bimodal distribution listeners are claimed to use to establish allophonic categories is truly bimodal in naturally-occurring contexts. Many allophonic relationships that were previously characterized as involving complementary distribution are better conceived of as existing on a continuum, with binary distribution as a tendency, rather than an absolute. This may particularly hold for learners who are exposed to cross-dialectal variability. For example, Recasens and Espinosa (2005) show that the degree of darkness found in /l/ allophones varies across dialects of Catalan. Carrasco et al. (2012) found a similar degree of variability in the voiced stops across different dialects of Spanish. Thus, what has often been understood as complementary distribution may in fact be better explained as dialect-dependent in degree and extension.

It is important that future research consider more closely how adult second language learners deal with variability in the speech stream and how language experience, proficiency and use interact with this. As Barlow's study reveals, it is not enough to simply predict how L2 sounds will assimilate into native language categories based upon target language and native language categories. It is necessary to consider the context of the sounds and the experience language learners bring to the task. Related to this is a need for research on how variability affects word processing, rather than merely perception of individual sounds. Indeed, recent work on cross-linguistic phonemic perception has revealed an important effect for task demands on L2 speech perception (Sebastián-Gallés and Díaz, 2012), and speech segmentation (Shea and Renaud, 2014). Further work is necessary to determine precisely how allophonic information is represented by L2 learners. For example, we might ask if lexical processing by second language learners is slowed down by mismatched allophones, or do they merely ignore it and consider it to be noise? Research shows that L2 learners are sensitive to context when hearing target-language allophones and when L2 listeners are exposed to allophonic variants outside of their expected contexts, processing is interrupted (Shea and Curtin, 2011).

In native language acquisition, researchers have been addressing issues of variability for quite some time and we need more research to help us understand how adult second language learners confront the same challenges. Evidence seems to be accumulating that outside the laboratory, the same distributional learning mechanism that allows infants to create phonetic categories during the first year of life may not afford adults acquiring a second language the same degree of success (Wanrooij et al., 2014). However, as stated above, adult learners can benefit from explicit instruction that can help them learn from regular, conditioned variability in the speech stream.

## Conflict of interest statement

The author declares that the research was conducted in the absence of any commercial or financial relationships that could be construed as a potential conflict of interest.
